# Liposome-mediated gene delivery: A comprehensive review of biophysical parameters, lipid composition and targeting strategies

**DOI:** 10.5599/admet.3065

**Published:** 2025-12-02

**Authors:** Erfan Jafari Saleh, Mohammad Satari, Behnam Hajipour-Verdom

**Affiliations:** 1 Department of Biology, Faculty of Sciences, University of Malayer, Malayer, Iran; 2 Department of Biotechnology, Faculty of Interdisciplinary Science and Technology, Malayer University, Malayer, Iran; 3 Integrative Oncology Department, Breast Cancer Research Center, Motamed Cancer Institute, Academic Center for Education, Culture and Research (ACECR), Tehran, 1517964311, Iran; 4 Department of Biophysics, Faculty of Biological Sciences, Tarbiat Modares University, Tehran, 14115-154, Iran

**Keywords:** Cationic lipids, non-viral vector, gene therapy, active targeting, transfection

## Abstract

**Background and purpose:**

Gene therapy has emerged as a transformative strategy for treating genetic and acquired disorders, but its clinical success relies heavily on the development of safe, efficient, and target-specific delivery vectors.

**Experimental approach:**

The paper systematically analysed published evidence on liposome composition, physicochemical behaviour, targeting strategies, and their applications in delivering diverse nucleic acids, with emphasis on the structure-function relationships of lipid components and the impact of biophysical parameters on transfection efficiency.

**Key results:**

Current findings demonstrate that the molecular architecture of cationic, neutral, and anionic lipids, particularly variations in head groups, linkers, and hydrophobic tails, strongly dictates liposome stability, cellular uptake, and cargo release. Biophysical attributes such as vesicle size, zeta potential, membrane fluidity, fusion capacity, and PEGylation were identified as major determinants of in vivo fate. Active targeting through ligands, including antibodies, peptides, folate, and aptamers, enhances cell-specific delivery, while combinatorial approaches with physical enhancement techniques such as sonoporation and electroporation further improve nucleic acid transport.

**Conclusion:**

By integrating structural, functional, and application-based insights, this review highlights key design principles for optimizing next-generation liposomal vectors, although challenges remain in achieving consistent in vivo performance and clinical translation. The work advances the field by offering a unified framework to guide rational engineering of liposomal platforms for gene therapy.

## Introduction

The rising prevalence of genetic disorders and cancers globally has underscored the need for newer therapeutic approaches 7. Gene therapy holds great promise due to its ability to target and modify genetic material at the cellular level, offering the prospect of a cure for diseases that are difficult or impossible to treat with conventional pharmacological therapies [3,4]. The majority of such diseases are caused by gene-level mutations, either congenital or acquired over time, that remain incurable with conventional drugs [5]. Therapeutic technology advances have enabled the development of strategies to edit, insert, delete, replace, or silence individual genes [6]. Successful delivery of genetic material and editing tools into primary cells, which are then delivered into target organs, is what successful gene therapy hinges on [7]. Therefore, the selection of an efficient and safe method of gene transfer is paramount [8,9].

Liposomes are neutral or charged colloidal drug and genetic material delivery vectors, which are prepared as unilamellar or multilamellar vesicles of 25 nm to 50 μm in diameter. They primarily consist of phosphorlipids, cholesterol, and an interior aqueous space [10,11]. The effectiveness of liposome-mediated transfection depends on biophysical parameters, including vesicle size, number of lipid bilayers, liposome fluidity, lipid phase separation, and surface charge [12]. Furthermore, every part of the phospholipid, such as the head group, tail, and body, plays a significant role in these factors [13]. Although various cationic polymers-notably polyethyleneimine (PEI) and spermine demonstrate specific merits for gene delivery, cationic liposomes collectively offer a more advantageous profile for clinical translation. PEI is widely recognized for its potent "proton-sponge" effect, which promotes efficient endosomal escape, a characteristic we have effectively utilized in previous studies for targeted drug delivery [14] and nucleic acid delivery [15]. Similarly, spermine is effective in our earlier work, facilitating gene transfer with appreciable efficiency owing to its strong nucleic acid condensation capacity [16]. In contrast, cationic liposomes are generally favoured due to their superior biocompatibility, reduced cytotoxicity, formulation simplicity, and consistently higher transfection efficiency-attributes that collectively underscore their broader utility and popularity in gene therapy applications [17]. On the other hand, it’s still less effective than viral vectors [18]. Liposomes are more effective and popular than other chemical-based vectors for effective gene delivery and gene therapy [19].

Gene delivery methods can be classified into viral, physical, and chemical strategies [20]. Viral vectors, developed by replacing pathogenic genes in viruses with therapeutic genes, remain widely used due to their naturally evolved genome-delivery properties [21]. Both of these vectors, chemical and viral, can be combined with various physical methods, such as microinjection, magnetofection, mechanical massage, hydrodynamic injection, sonoporation, laser-mediated delivery, biolistics, electroporation, thermal methods, and ultrasound-assisted delivery, which we will discuss in the continuation of this review [22].

## Gene therapy

Gene therapy is a milestone in the ongoing quest to cure, and even eliminate, many diseases [23,24]. Gene therapy encompasses some general strategies: gene silencing, gene replacement, and gene editing [24,25]. Gene silencing uses small interfering RNA (siRNA), short hairpin RNA (shRNA), and microRNA (miRNA) to reduce gene expression at the mRNA level [24,26]. Gene replacement therapy delivers functional genes, typically via plasmids or viral vectors, to supplement or replace protein expression [27]. In addition, gene editing directly targets specific mutations using engineered nucleases, such as zinc finger nucleases (ZFNs), transcription activator-like effector nucleases (TALENs), and the CRISPR/Cas system [28]. In continuation, for effective delivery of these proteins into target cells, vectors are required to protect them from enzymatic degradation and immune cell recognition [29].

Gene therapy has been explored for a wide variety of diseases, but the safe and effective delivery of genetic material remains the central challenge [25]. To be effective in gene therapy, the delivery vector must protect DNA molecules, hydrophilic and physiologically large with their negatively charged phosphate backbone, against nucleases for degradation and enhance cellular uptake [30]. Delivery systems, particularly vectors, are required because naked DNA cannot effectively penetrate cell membranes or resist enzymatic degradation [31].

### Gene delivery barriers

One of the most noticeable aspects of an effective gene therapy is the bypassing of gene delivery barriers, which can be categorized into intracellular and extracellular barriers [32], as shown in Table 1. The cell membrane is a prominent intracellular barrier. It consists of different proteins, phospholipids, cholesterol, and saccharides that play a significant role in preventing exogenous nucleic acids from entering the cell membrane [33]. Endocytosis is one of the most effective mechanisms for cellular uptake [34]. However, following endocytosis, lysosomes, which are charged with hydrolytic enzymes, pose another barrier by degrading the internalized genetic cargo [35].

**Table 1.** Intracellular and extracellular barriers affecting nucleic acid delivery.

Type	Barrier	Description
Intracellular	Cell membrane	Prevents entry of DNA due to charge and size [33]
Intracellular	Nucleus entry	Cargo must have an importin to be able to enter the nucleus [36]
Intracellular	Lysosomal degradation	Degrades internalized genetic material [35]
Extracellular	Circulating stability	Serum nucleases (*e.g.* DNase I) degrade naked DNA. Stabilization: PEGylation, nanoparticle encapsulation [37]
Extracellular	Administration route	Challenges vary by route (*e.g.* IV: serum clearance; IM: tissue diffusion; oral: GI degradation) [30]
Extracellular	Immune system	Recognizes and eliminates foreign particles [6]

Extracellular barriers also significantly contribute to in vivo gene delivery [30]. One of the largest challenges is the stability of the vector in blood, which contains a variety of negatively charged serum proteins and nucleases that can degrade nucleic acids [37]. To avoid this, gene delivery vectors are often designed to shield nucleic acids, either by encapsulation or by modification of surface epitopes, for instance, viral capsid proteins, to reduce immune recognition [27]. Pharmacokinetic properties like circulation half-life and systemic stability play significant roles in vector design [6].

The extracellular matrix acts as a physical barrier, and immune cells like liver Kupffer cells and splenic macrophages actively remove extracellular DNA-containing particles from the circulation [6]. Endosomal entrapment is the major intracellular barrier, where DNA degradation may occur before reaching the nucleus [38]. Endosomal escape mechanisms include membrane fusion, proton-sponge effect, and the use of pore-forming peptides [39]. Lipoplexes, for instance, have been shown to fuse with endosomal membranes, and cationic polymers such as PEI and fusogenic lipids such as cholesterol and DOPE facilitate endosomal release [7]. Once internalized, the vector must successfully navigate the cytoplasm and deliver its cargo from the endosome in sufficient time to facilitate nucleus import [36]. Delivery of genetic material into the nucleus is of primary importance to therapeutic effectiveness [36].

### Mechanism of liposome gene delivery

**Figure 1.**
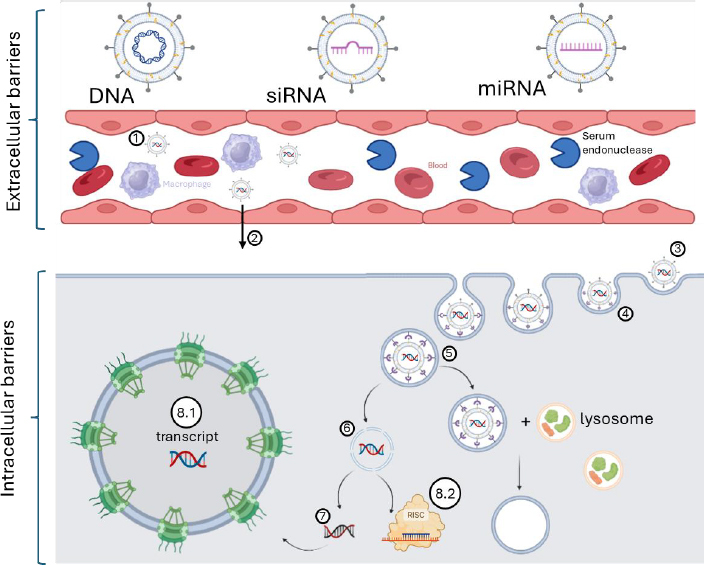
The schematic of key steps in liposome-mediated gene therapy. 1 - The process begins with the administration of liposomes into the bloodstream. 2 - The liposomes then extravasate from the blood vessels into the target tissue, 3 - bind to specific receptors, and are 4 - internalized via endocytosis. 5, 6 - Subsequent endosomal escape, mediated by mechanisms such as the sponge effect, fusogenic lipids, or penetration peptides, protects the cargo from lysosomal degradation and enables its release. 7, 8.1 - The cargo can then either enter the nucleus or 8.2 - perform its function in the cytoplasm. The figure was created with BioRender.com [42]

Liposomes are first administered as vectors in biological models, such as animals, humans, or other systems [30]. The goal of administration route selection (*e.g.* intravenous, intramuscular, or intratracheal) is to deliver the vector optimally to target cells, which may be located in specific tissues or blood vessels [6]. In the case of vascular administration, the vectors must contend with serum proteins and blood components [37]. Subsequently, liposomes extravasate from blood vessels into target tissues via active or passive targeting [2]. Following extravasation, they bind to specific cell surface receptors that stimulate endocytosis [31,38] (Figure 1).

During cellular uptake, the endosomal vesicles containing the liposomal vectors undergo acidification as lysosomes begin to merge with them [35]. As the vesicle pH decreases, amine groups on the liposomes absorb H⁺ ions [39]. To maintain ionic balance, the vesicle then takes up Cl^-^ ions, which enter accompanied by water molecules, ultimately causing the vesicle to burst - a phenomenon known as the proton sponge effect [40]. Alternatively, fusogenic lipids represent another important escape mechanism; as the vesicle pH continues to drop, these lipids become activated, facilitating cargo release into the cytoplasm [7]. In some cases, cell-penetrating peptides bind to liposomes and, upon pH reduction, form pores in the vesicle membrane [41]. However, in most instances, if escape mechanisms fail, lysosomes fully merge with the vesicle, activating pH sensitive enzymes that degrade the cargo [35].

## Liposome composition and structure

**Figure 2.**
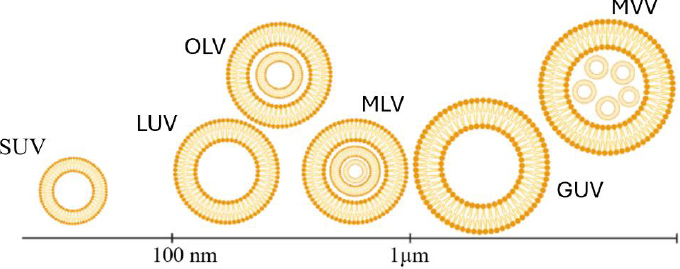
Classification of liposomal vesicles based on lamellar structure and size. The figure depicts various types of liposomal vesicles, including: OLV (oligolamellar vesicles), LUV (large unilamellar vesicles, ~100 nm), MLV (multilamellar vesicles, ~1 μm), GUV (giant unilamellar vesicles).

It has been well known that liposome characteristics are highly dependent on lipid composition and structure, which include head, tail, and body [43]. Also, all parts of lipids have special roles that will be discussed in continuation [44]. The head group of phospholipids plays a prominent role in phospholipid charge [45]. The lipid-based vector body consists of the connecting tail and head group [46]. Tails are essential for the rigidity and leak resistance of the vector [47]. Nanoliposomes, which are self-assembled phospholipid vesicular structures, vary from unilamellar to multilamellar structures, varying from unilamellar vesicles (ULVs), oligolamellar vesicles (OLVs), multilamellar vesicles (MLVs), and multivesicular liposomes (MVLs)-with varying effects on gene delivery efficiency [48,49] (Figure 2).

### Cationic lipids

Contemporary research classifies cationic lipids into four fundamental structural components that govern their functionality [50]. The hydrophobic core typically features either saturated alkyl chains (C12-C20) or cholesterol derivatives [51], with these moieties predominantly linked via robust ester or ether bonds. Serving as the molecular scaffold, the backbone structure most commonly employs a glycerol framework, although innovative designs have incorporated amino acid residues and aromatic systems. The cationic headgroup domain, crucial for nucleic acid complexation, predominantly utilizes quaternary ammonium groups that have demonstrated exceptional delivery efficiency. Researchers have also explored diverse cationic alternatives, including polyamine chains, guanidinium units, heterocyclic systems, and biomimetic amino acid/peptide conjugates, all of which show promising gene delivery capabilities [52].

### Monovalent cationic lipids

**Figure 3.**
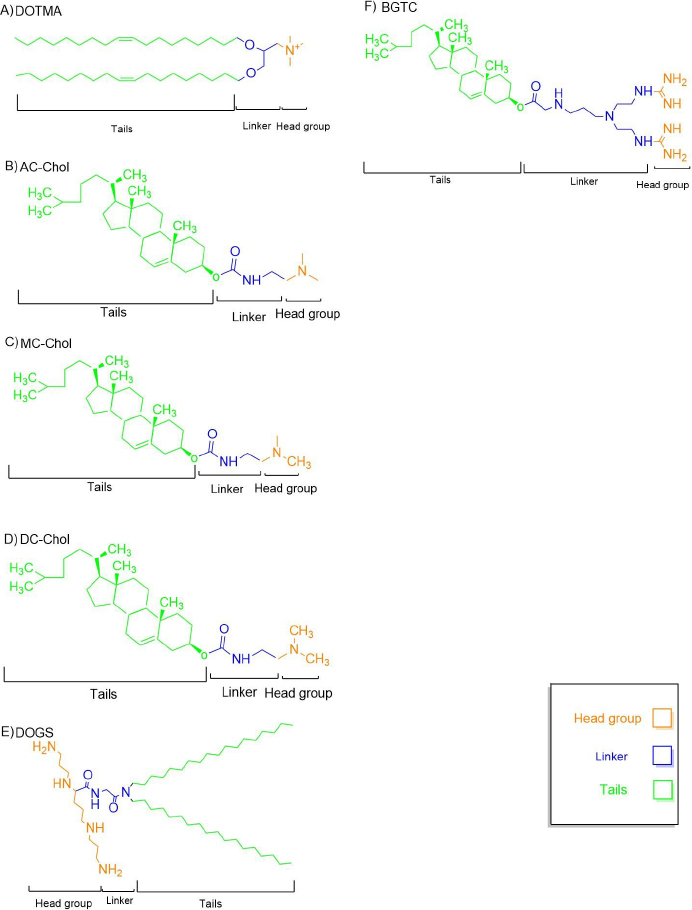
Chemical structures of representative cationic lipids used in gene delivery. The molecular components of each lipid are categorized into three regions: hydrophobic tails (green), linker (blue), and cationic head group (orange). Structures shown include: (A) DOTMA, (B) AC-Chol, (C) MC-Chol, (D) DC-Chol, (E) DOGS, and (F) BGTC. These structures differ in tail saturation, head group type, and linker chemistry, which influence their biophysical properties and transfection efficiency.

Monovalent cationic lipids have a single positively charged headgroup, which interacts electrostatically with negatively charged nucleic acids. DNA binding by the vector requires a headgroup capable of maintaining a positive charge at physiological pH. The charge is most often located on amino groups, as is the case for the 'early' vectors of Figure 3. However, other charged groups have since been employed. Thus, before dealing more extensively with amino-based head groups, it is worth briefly considering the other elements shown capable of binding plasmids. Phosphonium groups as well as arsonium groups (which, unlike arsenic(III) compounds, are not toxic) have been used to convey the positive charge of cationic lipids, which incorporate phosphonate linkers (as in natural phospholipids) [53,54]. The order arsenic > phosphorous > nitrogen was observed in vitro testing of the analogues, with the phosphonium and arsonium vectors being less cytotoxic than the ammonium-based lipid [55]. Further, cationic phosphonolipids were found to be effective in mouse lungs in vivo after intratracheal as well as intravenous administration [56].

#### DOTMA

N-[1-(2,3-dioleyloxy)propyl]-N,N,N-trimethylammonium chloride, or DOTMA, is one of the first cationic lipids synthesized and commercially available for gene delivery (Figure 3A). It has two unsaturated oleoyl chains (C18: Δ9) linked by an ether bond to a three-carbon glycerol backbone, and the cationic head group is a quaternary amine. Compared with other gene transfer methods in the late 1980s, DOTMA was found to be up to 100-fold more efficient at gene delivery than methods such as DEAE-dextran coprecipitation or calcium phosphate [19].

DOTMA was commercialized as Lipofectin, which was mixed with DOPE at a 1:1 ratio to maximize transfection efficiency. As Lipofectin was still being optimized, researchers began modifying DOTMA to reduce toxicity and improve efficiency across the three main parts of the lipid head group, linker, and hydrocarbon chains [57-59].

#### DOTAP

[1,2-bis(oleoyloxy)-3-(trimethylammonio)propane], or DOTAP, was synthesized by Leventis and Silvius in 1990 [60]. It is structurally similar to DOTMA but differs in that ester bonds, rather than ether bonds, link the oleoyl chains to the glycerol backbone. Ester bonds were believed to render DOTAP biodegradable, thereby reducing cytotoxicity. However, it was discovered that the transfection efficiency and cytotoxicity of DOTAP/DOPE preparations were comparable with those of DOTMA/DOPE complexes [61].

#### DC-Chol

3β[N-(N',N'-dimethylaminoethane)-carbamoyl]cholesterol, or DC-Chol, was synthesized by Gao and Huang in 1991 [18] (Figure 3D). DC-Chol contains a cholesterol moiety attached via an ester bond to a hydrolysable dimethylethylenediamine. Cholesterol was chosen for its biocompatibility and lipid-stabilizing effects. Unlike cationic liposomes that are composed of fully charged quaternary amines (*e.g.* DOTMA and DOTAP), DC-Chol contains a tertiary amine that is half-charged at physiological pH 7.4, which is thought to reduce lipoplex aggregation and facilitate transgene expression. The reduced overall charge of DC-Chol liposomes also favours DNA dissociation, which is a prerequisite for efficient transfection [62,63].

### Multivalent cationic lipids

#### DOSPA

2,3-dioleyloxy-N-[2(sperminecarboxamido)ethyl]-N,N-dimethyl-L-propanaminium trifluoroacetate, or DOSPA, is a cationic lipid derivative of DOTMA. The DOSPA structure is analogous to that of DOTMA, except for a spermine group appended to the hydrophobic chains via a peptide bond. In a 3:1 ratio with DOPE, DOSPA is sold commercially under the name Lipofectamine. The spermine functional group enables DNA condensation in liposomes, most likely due to its multiple ammonium groups, which can interact with DNA bases via hydrogen bonds and wrap around the major groove to interact with opposite-strand complementary bases [64].

#### DOGS

Di-octadecyl-amido-glycyl-spermine, or DOGS, is a structural analog of DOSPA (Figure 3E), but DOGS has saturated alkyl chains, and they are linked to the head group via a peptide bond, without the quaternary amine. DOGS, which is marketed as Transfectam, has been found to give transgene expression levels over 10 times higher than that using calcium phosphate transfections [65]. DOGS is poorly cytotoxic but efficiently condenses DNA due to the close interaction between the spermine head group and DNA. DOGS also buffers the endosomal compartment and protects the delivered DNA from degradation by pH-sensitive nucleases [66].

### Neutral and helper lipids

Lipid formulations for gene delivery typically include a combination of charged lipids and neutral helper lipids such as dioleoylphosphatidylethanolamine (DOPE) or dioleoylphosphatidylcholine (DOPC) [57,58] (Table 2).

**Table 2.** Functions and structural features of common helper lipids in nanocarrier formulations.

Helper lipid	Function	Structure	Comments
DOPE	Endosomal escape, fusogenicity	Unsaturated, small headgroup	Forms inverted hexagonal phase at acidic pH
DOPC	Membrane stability	Zwitterionic, bulky choline	Less efficient than DOPE
Cholesterol	Membrane packing, structural integrity	Rigid steroid ring	Enhances stability and uptake
PEG-lipids	Stealth behaviour	Hydrophilic polymer	Prolongs circulation, reduces immune clearance

DOPE is the most widely used of these neutral helper lipids [67]. Experiments demonstrated that DOPE is more effective at transfection than DOPC in most cell types because it can adopt an inverted hexagonal packing arrangement at low pH [68]. This inverted hexagonal structure differs from the lamellar packing observed in most DNA/lipid complexes and resembles a honeycomb of tube structures that trap DNA within the tubes via electrostatic forces [69]. Studies show that the hexagonal structure induces the effective release of DNA from endosomal vesicles by destabilizing their membranes [70-73].

Cholesterol (Chol) is another important helper lipid that helps lipoplex function [74]. As a neutral amphiphile, Chol promotes membrane packing and structural integrity without direct interaction with NAs [72]. Being present in lipoplex preparations enables it to interact with both plasma and endosomal membranes, thereby increasing transfection efficiency [75].

### Anionic lipids

Gene delivery with anionic lipids is generally inefficient. The negatively charged head groups of anionic lipids repel the phosphate backbone of DNA, preventing effective DNA compaction [39]. Despite this deficiency, anionic liposomes have become of interest as promising gene delivery vehicles due to the disadvantages of cationic liposomes, like inactivation by serum, instability in storage, and in vitro and in vivo cytotoxicity [76, 77]. DNA-containing liposomes can be assembled by anionic lipids using divalent cations, which neutralize electrostatic repulsion between negatively charged lipids and promote assembly of lipoplexes [78]. Common anionic lipids are naturally occurring phospholipids in cellular membranes, for instance, phosphatidic acid, phosphatidylglycerol, and phosphatidylserine [79]. The presence of phospholipids in natural cell membranes is associated with compromised clearance and macrophage phagocytosis when lipoplexes carry a net negative surface charge. This characteristic indicates enhanced biocompatibility and reduced immunogenicity, making anionic liposomes an attractive option for gene delivery [80].

### Detailed structural components of lipids

#### Head group

Cationic lipid polar headgroups primarily facilitate the binding of anionic nucleic acids (NAs) through electrostatic interactions, forming stable lipoplex complexes. The structural characteristics of these headgroups, such as size, charge density, and chemical composition, significantly impact lipoplex stability, NA condensation, cellular membrane interactions, and endosomal escape efficiency, thereby playing a pivotal role in transfection efficacy [81]. Common polar headgroup classes include quaternary ammonium salts, primary, secondary, and tertiary amines, guanidinium groups, heterocyclic compounds, and hybrid structures (Table 3). Advances in lipid design have expanded this repertoire, with biomacromolecular headgroups enabling multifunctional delivery systems with improved properties [82].

**Table 3.** Chemical classes of cationic lipid headgroups and their functional characteristics.

Headgroup type	Examples	Properties	Advantages	Disadvantages
Quaternary ammonium	DOTMA, DOTAP, DDAB	Permanently charged at pH 7	Strong DNA binding	Can be cytotoxic
Primary/secondary/tertiary amines	DODAP, CTAP	Weak bases, pH-responsive	Endosomal escape	Lower binding affinity
Polyamines	DOGS, DOSPA	Multiple amines	Strong condensation	Possible aggregation
Guanidinium	BGSC, BGTC, DSGLA	Charge delocalization	Strong binding	May hinder release
Heterocyclic	Imidazole, pyridine	Amphoteric	Efficient endosomal escape	Complex synthesis

Multivalent cationic lipids outperform monovalent counterparts in transfection efficiency due to their enhanced ability to bind and condense NAs while protecting them from intracellular degradation [83]. The NA-binding efficiency of these headgroups is pH-dependent, as protonation governs electrostatic interactions. The acid dissociation constant (p*K*_a_) of the conjugate acid determines the protonation state: headgroups with higher p*K*_a_ values remain cationic at physiological pH, promoting NA binding, while deprotonation at pH values above the p*K*_a_ reduces binding affinity. Protonatable headgroups also provide buffering capacity, facilitating endosomal escape via the proton sponge effect. In the acidic endosomal environment (pH 5.5 to 6), weakly basic headgroups protonate, inducing chloride influx, osmotic swelling, and vesicle rupture, which enhances NA release into the cytosol [40]. Given that inefficient endosomal escape is a major barrier in lipoplex-mediated gene delivery, buffering lipids are often favoured over strongly cationic, non-buffering alternatives. Thus, rational headgroup design, optimizing protonation behaviour, charge density, and buffering capacity, is critical for developing efficient cationic lipid-based gene delivery systems to maximize transfection efficiency and therapeutic outcomes.

#### Quaternary ammonium compounds

Quaternary ammonium (NR⁺) compounds are organic cations with a permanently charged nitrogen atom covalently linked to four organic substituents. These headgroups maintain a stable positive charge at physiological pH, enabling robust NA binding and sufficient aqueous solubility [84]. Consequently, quaternary ammonium groups are among the most widely adopted polar headgroups in cationic lipid design [85]. Prominent examples of gene delivery vectors include 1,2-di-O-octadecenyl-3-trimethylammonium propane (DOTMA), 1,2-dioleoyloxy-3-[trimethylammonium]-propane (DOTAP), dimethyldioctadecyl ammonium bromide (DDAB) [5], and cetyltrimethyl ammonium bromide (CTAB) [54]. Notably, DOTMA, the first synthesized quaternary ammonium-based lipid, remains a cornerstone in this category.

#### Primary, secondary, tertiary amines and polyamines

Amines, categorized as primary, secondary, or tertiary based on the number of organic substituents on the ammonia molecule, are versatile weak bases with acid-base properties influenced by substitution patterns. Secondary amines (p*K*_a_ ~ 10.8) are slightly more basic than primary amines (p*K*_a_ ~ 10.6), while tertiary amines (p*K*_a_ ~ 9.8) exhibit reduced basicity due to steric hindrance [86]. At physiological pH, amine headgroups are typically neutral or weakly cationic, extending circulation half-lives but reducing NA binding strength compared to quaternary ammonium groups [87]. The transfection efficacy of amine-based lipids varies [88].

Polyamine-containing lipids are highly effective for gene delivery. For instance, CTAP, a penta-amine derivative, demonstrated 100-fold higher transfection efficiency than conventional DC-Chol/DOPE formulations due to the high charge density of fully protonated polyamines at physiological pH, enabling efficient NA neutralization and condensation [89]. Structural configuration is critical, with linear polyamines outperforming branched, T-shaped, or globular variants in NA condensation and transfection [89] with DOGS, inspiring spermine-based vector designs [87]. Although branched polyamines offer conformational stability [90], their transfection efficiency is generally lower than that of linear analogues [90].

#### Guanidinium headgroup

Guanidine, prevalent in arginine residues of DNA-binding histones, exhibits strong NA binding due to its near-complete protonation to guanidinium cations across a wide pH range, facilitated by charge delocalization across three nitrogen atoms [91,92]. The guanidinium group supports bidentate hydrogen bonding with anionic phospholipids in cell membranes, enhancing lipoplex internalization [93]. However, excessive binding affinity may impede NA release, potentially reducing gene delivery efficiency [94,95]. Multivalent derivatives combining guanidinium with pyridinium or amine groups have since improved transfection efficiency and reduced cytotoxicity compared to monovalent counterparts [96]. For example, DSGLA, incorporating guanidinium and lysine residues, showed superior siRNA binding compared to DOTAP [97]. Amidine derivatives, formed by substituting a guanidine amine with an R group, offer tuneable basicity (p*K*_a_ 5 to 12) and consistent protonation at the imino nitrogen, with the TRX gemini surfactant outperforming Lipofectamine in transfection efficiency with lower cytotoxicity [98].

#### Heterocyclic headgroups

Heterocyclic compounds, such as pyridine and imidazole derivatives, are widely used as cationic headgroups in gene delivery systems due to their amphoteric properties. Imidazole, with approximately 100-fold greater basicity than pyridine, protonates in acidic environments to form pyridinium or imidazolium ions with delocalized positive charges, optimizing NA binding-release dynamics through pH-sensitive behaviour [99,100]. Cyclen-based lipids with imidazole headgroups (p*K*_a_ ~ 6.0) [99] align with endosomal pH, promoting efficient transfection via the proton sponge effect [82].

### Body (linker)

In cationic lipids, the linker moiety serves as a critical structural bridge connecting the hydrophilic headgroup to the hydrophobic tails, significantly influencing the physicochemical properties and biological performance of lipoplexes. This linkage governs molecular stability, biodegradability, cytotoxicity, and transfection efficiency [101]. The linker’s chemical properties-such as charge distribution, length, and steric constraints-modulate the conformational flexibility of the amphiphile, thereby affecting the spatial arrangement of functional groups, nucleic acid (NA) binding affinity, and overall gene delivery efficacy [102].

Linkers are typically classified by their core chemical structures, with common types including ether, ester, carbamate, and amide bonds (Table 4). Recent advancements have focused on designing stimulus-responsive linkers that undergo controlled structural changes in response to environmental cues, such as pH variations, redox potential, or enzymatic activity [103]. The following sections detail key developments in linker design and their impact on gene delivery applications.

**Table 4.** Overview of linker types and their roles in cationic lipid design.

Linker type	Example lipids	Properties	Biodegradability	Role in gene delivery
Ether	DOTMA	Chemically stable	Poor	High transfection, low degradability
Ester	DOTAP	Acid-/enzyme-labile	Good	Biodegradable
Carbamate	DC-Chol	pH-sensitive	Good	Efficient and low toxicity
Amide	DOGS	Acid-labile	Moderate	Stable, long-term transfection
Disulfide	DOGSDSO	Redox-sensitive	High	Controlled NA release
Miscellaneous	Hydrazone, ketal	Stimuli-sensitive	Variable	Stimuli-triggered release

#### Ethers

Ether linkages, characterized by an oxygen atom bridging two alkyl groups (Figure 3A), confer high stability to cationic lipids but pose challenges for biodegradability. Comparative studies demonstrate that ether-linked lipids, such as DOTMA, exhibit up to 10-fold higher transfection activity than ester-linked lipids, such as DOTAP, in the absence of helper lipids [103]. Other ether-containing formulations similarly outperform ester- and carbamate-based lipids in gene delivery [104]. However, the chemical stability of ether bonds, which requires harsh conditions for cleavage, limits their metabolic degradation and may increase cytotoxicity risks [105].

#### Esters

Ester linkages, formed by substituting the hydroxyl group of a carboxylic acid with an O-alkyl (alkoxy) group (Figure 3B), are highly water-soluble due to their hydrogen bond acceptor properties. These bonds are susceptible to acid-catalyzed hydrolysis in acidic intracellular environments and enzymatic cleavage by endogenous esterases or lipases, rendering them readily biodegradable [29]. DOTAP, an early ester-containing cationic lipid (Figure 3B), demonstrated high transfection activity [106], leading to its use in preclinical and clinical lipoplex formulations [107]. However, the chemical instability of ester bonds can reduce delivery efficiency [65].

#### Carbamates

Carbamate (urethane) linkers provide greater stability than simple esters, resulting in lipoplexes with excellent transfection efficiency and low cytotoxicity [108]. DC-Chol, a representative carbamate-containing cationic lipid (Figure 3C), exemplifies these properties. Carbamate linkers are hydrolysed in the acidic endosomal compartment and cleaved by intracellular esterases, ensuring biodegradability [109]. Carbamate-containing lipids outperformed ether- and ester-based analogues, as well as Lipofectamine, while exhibiting lower cytotoxicity [110]. Similar findings were observed with carbamate-containing gemini quaternary ammonium lipids [58]. Due to their high transfection efficiency and stability in extracellular fluids, carbamate-linked lipids have advanced to gene therapy clinical trials [111].

#### Amides

Amide linkages, commonly found in peptides and proteins (Figure 3D), are exemplified by DOGS, a cationic lipid with an amide linker connecting a spermine headgroup to a saturated alkyl chain [112]. DOGS-based lipoplexes exhibit superior transfection efficiency compared to DOTAP, partly due to their pH-buffering capacity [112]. Amide bond hydrolysis in acidic endosomal environments follows a mechanism similar to esters, involving protonation of the carbonyl oxygen and nucleophilic attack by water, yielding a carboxylic acid and an ammonium ion [65]. Amide linkages are more stable and less cytotoxic than ester- or ether-based linkers, particularly in pH-sensitive systems. Their stability is attributed to intermolecular hydrogen bonding, which increases lipid melting points and structural integrity [39], confirming that amide-linked lipids are more stable and effective for gene transfection than ester-linked counterparts [113]. The positional orientation of the amide group relative to the cationic headgroup also influences transfection efficiency, with classical amide configurations, where the amine is proximal to the polar head, achieving efficiencies comparable to Lipofectamine 2000 across various cell types, whereas reverse isomeric amide bonds reduce efficiency due to increased molecular rigidity from Coulombic repulsion [114].

#### Miscellaneous

Less common acid-labile linkers, such as acetals/ketals and hydrazones, exhibit hydrolysis rates comparable to carbamates and vinyl ethers [115]. Ketal linkages, in particular, are highly sensitive to endosomal pH while remaining stable at physiological pH, making them ideal for circulation-stable, intracellularly responsive systems [39]. Acylhydrazone bonds also hydrolyse rapidly in acidic lysosomal environments [116]. Redox-sensitive disulfide (-S-S-) linkers enable spatially and temporally controlled lipoplex disassembly and NA release by responding to intracellular reducing agents like glutathione (GSH) [117]. Disulfide linkers are particularly effective for siRNA delivery, with cysteine-containing linkers enhancing structural stability and controlling cytoplasmic release [118]. Enzyme-cleavable linkers offer site-specific NA delivery regulated by target enzyme activity. For instance, the PEG-peptide-DOPE (PEG-PD) system disassembles upon peptide cleavage by matrix metalloproteinase-2 (MMP-2), an enzyme overexpressed at pathological sites [119]. Photosensitive linkers provide an alternative for stimulus-responsive gene delivery. UV-sensitive cationic amphiphiles, demonstrating that photolytic linker cleavage enabled endosomal NA release, achieving transfection efficiencies up to 20-fold higher than lipofectin [120].

Collectively, these findings highlight the critical role of linker chemistry in modulating lipoplex stability, cytotoxicity, and transfection efficiency. Biodegradable, stimulus-responsive linkers, particularly carbamates and disulfides, are particularly promising due to their ability to combine high transfection activity with low toxicity.

### Tails (hydrophobic region)

The hydrophobic tail moiety of cationic lipids is a critical determinant of lipoplex physicochemical properties, influencing phase behaviour, membrane fluidity, lipoplex stability, and cytotoxicity, as established through structure-activity relationship (SAR) studies [121]. These non-polar tails are broadly categorized into aliphatic chains or cyclic, steroid-like structures based on their structural features.

#### Aliphatic chains

Aliphatic tails, consisting of saturated or unsaturated hydrocarbon chains, significantly govern lipoplex performance. Factors such as chain length, degree of unsaturation, and the number of chains per lipid molecule are known to affect transfection efficiency [122], yet there is no universal agreement on the optimal tail configuration [19]. Generally, shorter saturated chains enhance transfection, with some studies indicating optimal performance for chains of 10-14 carbons [123]. However, other investigations, including our own, suggest a bell-shaped relationship, with peak efficiency at C14, diminishing for both shorter and longer chains [124].

The number of hydrophobic chains per lipid molecule remains a subject of debate. Double-tailed cationic lipids, such as DOTMA, DOTAP, DOPSA, DORIE, and DOGS, generally outperform their single-tailed counterparts, likely due to their ability to form stable aggregates in aqueous environments, which is influenced by molecular geometry and chemical properties [125]. Single-tailed lipids often form less stable, more cytotoxic complexes [126]. Notably, mixed formulations combining single- and double-tailed lipids can synergistically enhance transfection efficiency [127]. Unsaturated alkyl chains are widely recognized for enhancing transfection due to increased membrane fluidity and fusogenic properties [128]. The degree, position, and configuration of double bonds are critical [129]. While few studies directly correlate unsaturation with transfection efficiency, monounsaturated tails are generally most effective [122]. It was confirmed that C18 monounsaturated single-tailed lipids excel in DNA condensation and transfection. Additionally, the cis configuration of unsaturated chains consistently outperforms the trans configuration [130].

#### Cyclic (steroid-based) domains

Since the development of DC-Chol, a cholesterol-based cationic lipid, steroid-based transfectants have gained prominence [62]. Cholesterol is frequently utilized due to its rigidity, biodegradability, biocompatibility, and fusogenic properties [131]. For instance, synthesized cholesteryloxypropan-1-amine (COPA) and cholesteryl-2-aminoethylcarbamate (CAEC), which exhibited high siRNA delivery efficiency. Similarly, Bhattacharya’s group developed gemini-type cholesterol-derived cationic surfactants with transfection efficiencies comparable to single-tailed counterparts and Lipofectamine [132]. Beyond cholesterol, other steroid-based scaffolds, including vitamins, bile acids, cholestane, and lithocholic acid, have shown potential. Vitamin D_2_ and D_3_ analogues have yielded transfection agents comparable to DC-Chol [133], while α-tocopherol (vitamin E)-based lipids have demonstrated enhanced transfection activity. Bile acid-based lipids have also achieved success in gene delivery systems, albeit to a lesser extent [134].

Collectively, these findings highlight the importance of aliphatic and steroid-based hydrophobic domains in designing effective cationic lipid transfectants. Single-tailed cholesterol-based lipids and double-tailed monounsaturated aliphatic lipids consistently emerge as highly promising lipophilic motifs for gene delivery (Table 5).

**Table 5.** Overview of hydrophobic tail types and their roles in cationic lipid design

Tail type	Structure	Role	Key features	Transfection effect
Aliphatic chains	Saturated/unsaturated C14-C18	Membrane fluidity, fusion	Double tails improve stability	C14-C18 monounsaturated are optimal
Cyclic (steroidal)	Cholesterol, bile acids	Rigidity, biocompatibility	Enhances endosomal escape	DC-Chol widely used
Tail number	Single/double/multi-tail	Aggregation behaviour	Double-tail > single-tail	Stability and DNA condensation

### Helper lipids

The efficacy of cationic lipids in nucleic acid (NA) delivery is significantly enhanced when co-formulated with zwitterionic helper lipids, such as 1,2-dioleoyl-sn-glycero-3-phosphoethanolamine (DOPE) or 1,2-dioleoyl-sn-glycero-3-phosphocholine (DOPC). These co-lipids promote the assembly of supramolecular structures, improving lipoplex colloidal stability and enhancing interactions with cellular membranes [135].

DOPE’s structure, featuring a compact phosphoethanolamine headgroup linked via ester bonds to two unsaturated oleyl chains, imparts fusogenic properties that facilitate endosomal escape. In acidic environments (pH 4-5), DOPE-containing lipoplexes undergo conformational changes that destabilize the complex, promoting NA release [136,137]. These attributes have made DOPE a key component in commercial transfection reagents, such as Lipofectin (DOTMA:DOPE, 1:1 weight ratio) and Lipofectamine (DOSPA:DOPE, 3:1 weight ratio) [138].

In contrast, DOPC’s zwitterionic structure, with both an anionic phosphate and a cationic choline group, forms more stable lipoplex complexes but exhibits lower transfection efficiency compared to DOPE-based systems [139]. Comparative studies demonstrate that DOPE formulations enable faster endosomal trafficking and nucleus delivery of DNA, whereas DOPC-based lipoplexes are often retained in late endolysosomes, limiting their efficacy [140].

Cholesterol, a neutral amphiphile, is another critical helper lipid that enhances lipoplex function through distinct mechanisms. Cholesterol promotes membrane packing and structural stability without directly interacting with NAs. Its incorporation into lipoplex formulations enhances interactions with plasma and endosomal membranes, thereby improving transfection efficiency [140].

Collectively, these findings underscore the pivotal role of helper lipids in optimizing gene delivery systems. DOPE and cholesterol remain the most widely used due to their favourable membrane interaction properties, while ongoing research continues to explore optimal co-lipid combinations and innovative designs to further enhance transfection efficiency.

## Biophysical parameters influencing gene delivery

### Liposome size

The size of the liposomes is a factor in drug delivery. It directly influences the duration they remain in the bloodstream, as nanoparticles and macromolecules are removed by the renal system and the mononuclear phagocytic system (MPS) [141,142]. Nanoparticles smaller than 10 nm are rapidly excreted by the kidneys, while bigger particles are predominantly eliminated by the MPS [142]. Tumours, in contrast, exhibit leaky vasculature with widened endothelial pores that enable nanoparticles of appropriate size to accumulate in the tumour tissue but not normal organs, a phenomenon termed the enhanced permeability and retention (EPR) effect [143,144]. Comparative research on liposomes of different sizes has indicated that optimum extravasation across tumours is around 400 nm, whereas particles smaller than 200 nm are better in delivering drugs [145,146].

### Liposome charge

To prolong blood circulation, zwitterionic phosphatidylcholine (PC) liposomes are commonly used to minimize protein binding [147,148]. Conversely, incorporating negatively charged lipids accelerates liposome clearance due to protein adsorption, while cationic lipids, typically used for nucleic acid delivery, can facilitate cellular uptake by condensing negatively charged nucleic acids [149]. However, cationic lipids are generally toxic to cells and are rapidly cleared from circulation [29]. Cationic liposomes are particularly effective for delivering negatively charged macromolecules, such as DNA and RNA, which are challenging to deliver passively into cells [150]. Additionally, they selectively target angiogenic endothelial cells in tumours and are considered a promising tool for brain drug delivery as they can cross the blood-brain barrier (BBB) [151]. However, the positive charge can lead to aggregation in the bloodstream [152].

### Liposome fluidity and rigidity

The structure of the lipid tail exerts a strong influence on the phase transition temperature (*T*_c_) of lipids, which governs their mechanical stability, lateral diffusion, and membrane permeability. The membrane's permeability is greatest near *T*_c_ due to the coexistence and dynamic interconversion of the fluid and gel phases [153]. This concept has been widely exploited in the design of thermally responsive liposomal drug delivery vectors [154]. Lipids with higher *T*_c_ values create more rigid membranes, whereas lower *T*_c_ lipids are more fluid. This mechanical property influences both cellular uptake as well as ECM penetration by liposomes [155]. Schroeder *et al.* [156] demonstrated that the internalization of pH-sensitive cationic liposomes in 4T1 breast cancer cells was enhanced with tail length: DSPC (18:0) > DPPC (16:0) > DMPC (14:0) [157]. Since tumours are encapsulated by a dense ECM, liposome deformability also influences their diffusion and retention. Moderately rigid DPPC-based liposomes exhibited improved penetration and retention in multicellular spheroids with a rich fibrous ECM [158].

### Lipid membrane fusion

Lipid membrane fusion is a critical process in numerous biological processes, but will not occur spontaneously [159]. Fusion only occurs when membranes are closely apposed and both electrostatic and hydration barriers are overcome. The process may be facilitated by metal ions, fusogenic lipids such as phosphatidylethanolamine (PE), and specific ligand-receptor interactions [160]. Kros *et al.* [161], for example, employed coiled-coil-peptides for selective liposomal drug delivery, revealing enhanced cellular uptake via predominantly membrane fusion rather than endocytosis [162]. Membrane-fusogenic liposomes (MFLp) that were functionalized with a cell-penetrating arginine peptide and a pH-sensitive PEG coating. After the shedding of the PEG chains in the acidic tumour environment, fusogenic peptides were revealed, and rapid membrane fusion and cytoplasmic delivery of drug cargoes were enabled within 30 seconds, avoiding endocytosis

### Effect of lipid phase

In the presence of cholesterol, phospholipids can exist in a liquid-ordered (Lo) phase, as well as the more fluid liquid-disordered (Ld) phase, which is enriched in unsaturated lipid species. Certain ternary and quaternary lipid mixtures phase separate into Lo and Ld domains [150]. Stachowiak *et al* [163] investigated the use of phase-separated liposomes for enhanced cytoplasmic delivery. They prepared phase-separated liposomes composed of DOTAP/DPPC/cholesterol and found they demonstrated lipid transfer rates 8 to 10 times higher to giant unilamellar vesicles (GUVs) than their non-phase-separated counterparts. In cellular uptake experiments, phase-separated liposomes containing 5 mol.% DOTAP achieved 4-5-fold greater fusion efficiency with cells compared to homogenous liposomes, likely due to the concentration of DOTAP in distinct membrane domains.

### Role of PEGylation

Polyethylene glycol (PEG) modification, or PEGylation, is a widely utilized strategy to prolong liposome circulation by reducing plasma protein adsorption and thereby minimizing phagocytic clearance. Typically, PEG chains are anchored to liposomes using PEGylated lipids such as PEG-DSPE. Huang and colleagues demonstrated that PEGylated large unilamellar liposomes (~200 nm) exhibited a circulation half-life of approximately 5 hours, in contrast to less than 30 minutes for non-PEGylated counterparts [164]. The impact of PEG on liposome behaviour depends on both its molecular weight and surface density [165]. While PEGylation generally enhances circulation time, excessive PEG can adversely affect drug delivery performance by reducing cellular uptake and impairing endosomal escape [165]. Moreover, repeated administration of PEGylated liposomes may trigger the accelerated blood clearance (ABC) phenomenon [166].

## Strategies for targeted gene delivery

The success of gene therapy relies on the ability to selectively deliver therapeutic genes to a sufficient number of target cells [167,168]. This can be achieved through physical targeting and molecular biological targeting [169,170]. Physical targeting relies on the attachment of ligands to the delivery vehicle that bind to cell-surface receptors unique to the target cells [171,172]. Molecular biological targeting refers to the selective expression of a therapeutic gene in the target cell using a specific promoter [173,174].

### Active targeting

Actively targeted liposomes represent a third-generation advancement designed to enhance therapeutic selectivity and cellular uptake [175,176]. Unlike passive targeting, which relies on the enhanced permeability and retention (EPR) effect in tumour tissues, active targeting employs molecular recognition elements to facilitate selective binding to diseased cells, thereby promoting receptor-mediated endocytosis [177]. Actively targeted liposomes have been developed by incorporating ligands such as simple peptides, antibodies and their fragments, carbohydrates, nucleic acids, and vitamins, which recognize and bind to overexpressed receptors on tumour cells [178-180]. Overall, active targeting facilitates the precise localization of therapeutics at the site of action, enabling lower drug doses and reduced systemic toxicity [181].

### Targeting ligands

#### Antibodies (Immunoliposomes)

Antibodies are glycoproteins produced by the immune system that selectively recognize and bind to antigens [182,183]. By attaching antibodies to liposome surfaces, it is possible to create highly specific drug-loaded nanoparticles, so-called immunoliposomes [184]. This approach has shown promise for better targeted delivery of therapeutics; however, the results have been inconsistent [185,186]. Recently, experiments have shown that the utilization of only the Fab portion of the antibody can be more effective than the whole antibody [187-189]. Whole antibodies can lead to enhanced immunogenicity, larger particle sizes, and reduced efficacy compared to liposomes coupled with Fab fragments [188,190].

#### Peptides

Peptides, being short chains of amino acids, have long been known for their therapeutic potential [191]. Their unique structure enables them to modulate protein-protein interactions [192]. Due to their specificity and therapeutic activity, peptides have also been conjugated to liposomes for targeted drug delivery [193]. Diseased tissues often overexpress certain receptors (*e.g.* HER2 in breast cancer), and peptides can be designed to bind selectively to these receptors, enabling targeted delivery [194,195]. For instance, a cyclic arginine-glycine-aspartic acid-tyrosine-lysine peptide (cRGDyk) was used to target αvβ3 integrin, a receptor overexpressed in bone metastasis [196,197]. cRGDyk-conjugated liposomes exhibited more than threefold higher tumour cell internalization rates and were more than 88 % more cytotoxic than free drugs [198]. Despite their promise, challenges such as defining peptide density and storage stability remain [199,200].

#### Folate

Folate, or vitamin B9, plays a role in both the onset and growth of cancer [201,202]. Tumour cells that undergo rapid proliferation have a high requirement for DNA synthesis and are prone to overexpression folate receptors [203,204]. As a result, folate receptors have become an attractive target in cancer therapies [205]. Folate can be conjugated to liposomes to target these overexpressed receptors [206]. Numerous investigations have demonstrated that folate-conjugated liposomes exhibit increased cellular uptake and improved specificity in vitro [206,207]. However, challenges remain regarding their *in vivo* behaviour, as some studies show that intravenous administration is not much more effective at increasing drug uptake in tumours than control liposomes, and that poor drug unloading at the target site can compromise efficacy [208,209].

#### Aptamers

Aptamers are single-stranded RNA or DNA molecules that have a defined three-dimensional structure and the capacity to bind selectively to target molecules [210]. Hailed as "chemical antibodies," aptamers offer high specificity, stability, and low immunogenicity [211-213]. This selectivity makes aptamers a strong candidate for the development of targeted drug-delivery vectors [214,215]. Researchers have utilized aptamer-conjugated liposomes to deliver drugs to a wide variety of tissues, including cancer cells, corneal cells, and muscle tissue [212,216]. Compared with antibody-conjugated liposomes, aptamer-conjugated liposomes have shown better performance in some in vivo tests, stimulating growing interest in their use [216].

## Types of genetic cargo and delivery strategies

### DNA and mRNA delivery

Targeted and efficient delivery of therapeutic genetic material is a cornerstone of progress in cancer gene therapy [217]. Liposomes are a highly promising vehicle for delivering both DNA and mRNA [218]. Both are effectively stabilized and protected against nuclease degradation through complexation with cationic lipids that enable them to be internalized by target cells [219,220]. Encapsulation strategies not only protect DNA from enzyme degradation and immune recognition but also maintain the structural integrity of genetic material in transit [221]. Recent developments have also highlighted the potential of mRNA therapies, particularly cancer vaccines encoding tumour-specific antigens to elicit strong immune responses [222]. The rapid development and regulatory approval of mRNA vaccines during the COVID-19 pandemic have accelerated research into mRNA cancer vaccines [223].

### siRNA delivery

RNA interference (RNAi), as mediated by small interfering RNA (siRNA), enables post-transcriptional gene silencing by inducing target mRNA degradation or preventing its translation [224,225]. Typically, siRNAs are 21 to 23 nucleotides in length [226]. While it shows promise as a therapeutic agent, the clinical application of siRNA is limited by rapid nuclease-mediated degradation, uptake by immune cells, and poor tissue penetration [227]. To overcome these limitations, nanocarrier-mediated delivery vectors have been developed. Of these, liposomes have emerged as promising candidates, enabling siRNA entry into tumour cells through various endocytic processes, including clathrin-mediated endocytosis, macropinocytosis, and cholesterol-dependent membrane transport [228,229].

### shRNA delivery

Short hairpin RNAs (shRNAs) are another form of genetic agents used for cancer treatment [230,231]. As members of the short non-coding RNA (ncRNA) family, shRNAs behave similarly to siRNAs in repressing gene expression through RNA interference-mediated gene knockdown [232]. Following delivery into cells, usually via a vector, shRNAs are transcribed within the nucleus and subsequently form hairpins [232,233]. These hairpin RNAs are then exported to the cytoplasm, where the enzyme Dicer cleaves them into siRNA-like molecules, which are then loaded into the RNA-induced silencing complex (RISC) to guide the degradation or translational suppression of target mRNAs [233-235].

### MicroRNA delivery

MicroRNAs (miRNAs) are vital members of the non-coding RNA (ncRNA) family, typically 18 to 24 nucleotides in length, that regulate gene expression post-transcriptionally [236]. MiRNAs are involved in the regulation of critical cell processes, including apoptosis, cell growth, and metastasis [236-238]. Aberrant miRNA expression is responsible for a variety of diseases, notably cancer, and modulation of miRNA activity holds promise as a future therapeutic method [239,240]

## Enhancing delivery with physical methods

Physical methods are associated with employing physical strength to enhance the penetrability of the cell membrane, which aids in the delivery of the desired gene. The elemental superiority of this method is convenience and reliability, but a key disadvantage is the potential for cell injury during the process [241]. Generally, combining liposomes and physical gene delivery methods is highly recommended [242].

### Ultrasound (sonoporation)

This technique utilizes physically induced ultrasound forces to temporarily increase cell membrane permeability for the delivery of genetic material [243]. The primary mechanisms are cavitation (the creation and collapse of microbubbles), radiation pressure, and microstreaming, which generate transient pores in the membrane [244]. Sonoporation is non-invasive and safe, but the technique has low transfection efficiency and is often supported by other delivery vectors, such as liposomes, to improve outcomes [245].

### Magnetofection

Magnetofection is a method in which therapeutic genetic material is conjugated to magnetic nanoparticles and delivered to target cells using an external magnetic field [246]. Both magnetic fields and magnetic nanoparticles play pivotal roles in this technique, and these elements have found broad applications across various biological fields [247-250]. For in vitro applications, electromagnets are placed beneath the cell culture plate to attract the gene-magnetic nanoparticle complexes to the cells, thereby significantly enhancing uptake. This method is particularly useful for transfecting primary cells and other hard-to-transfect cell types [251].

### Electroporation

DNA can be transfected into any cell by applying controlled short electrical impulses [252]. An electric impulse is sufficient to transiently alter the structure of the cell membrane, creating pores that enable nanosized oligonucleotides and chemical compounds to enter the cell [253]. It is a cost-effective, quick, and highly reliable method that can be as effective as viral transfection. However, extensive optimization of conditions is required, as the cell death rate in cells subjected to this method is high [254].

### Laser (optoporation)

Light amplification by stimulated emission of radiation (LASER) assisted transfection, also called photo transfection or optoporation, relies on the concept that a laser pulse can transiently modulate cell membrane permeability [255,256]. The subsequent pore formation leads to an osmotic difference between the cytosol and the medium, thereby facilitating the entry of genetic materials into the desired cell [42].

## Conclusions

Liposomes stand as a versatile and powerful platform technology for DNA, mRNA, and siRNA delivery in the rapidly advancing field of gene therapy. Their remarkable tunability in terms of composition, size, charge, and surface modification allows for the rational design of vectors tailored to specific therapeutic needs. The ongoing exploration of novel lipid structures, particularly those with biodegradable linkers and pH-responsive headgroups, continues to push the boundaries of transfection efficiency while minimizing cytotoxicity. Furthermore, the integration of active targeting moieties and stimuli-responsive components is paving the way for highly specific, "smart" delivery systems capable of navigating complex biological barriers. While challenges related to in vivo stability, endosomal escape, and large-scale manufacturing persist, the synergistic combination of advanced liposomal formulations with physical enhancement techniques holds immense promise. As our understanding of the intricate interactions between nanocarriers and biological systems deepens, liposome-based vectors are poised to play an indispensable role in translating the potential of gene therapy into tangible clinical realities.

## Abbreviations

ABC: - Accelerated blood clearance
AC-Chol: - Amino cholesterol
BBB: - Blood-brain barrier
BGTC: - Bis(guanidinium)-tren cholesterol
CRISPR: - Clustered regularly interspaced short palindromic repeats
DC-Chol: - 3β-[N-(N′,N′-dimethylaminoethane)-carbamoyl]cholesterol
DEAE: - diethylaminoethyl
DOGS: - Di-octadecyl-amido-glycyl-spermine
DOGSDSO: - Di-octadecyl-amido-glycyl-spermine disulfide oxide
DOPC: - 1,2-dioleoyl-sn-glycero-3-phosphocholine
DOPE: - 1,2-dioleoyl-sn-glycero-3-phosphoethanolamine
DOSPA: - 2,3-dioleyloxy-N-[2(sperminecarboxamido)ethyl]-N,N-dimethyl-L-propanaminium trifluoroacetate
DOTAP: - 1,2-bis(oleoyloxy)-3-(trimethylammonio)propane
DOTMA: - N-[1-(2,3-Dioleyloxy)propyl]-N,N,N-trimethylammonium chloride
DPPC: - 1,2-dipalmitoyl-sn-glycero-3-phosphocholine
DSPC: - 1,2-distearoyl-sn-glycero-3-phosphocholine
EPR: - Enhanced Permeability and Retention
ECM: - Extracellular matrix
GUV: - Giant unilamellar vesicle
HSPC: - Hydrogenated soy phosphatidylcholine
LASER: - Light Amplification by Stimulated Emission of Radiation
Ld: - Liquid-disordered phase
Lo: - Liquid-ordered phase
LUV: - Large unilamellar vesicle
MFLp: - Membrane-fusogenic liposome
MLV: - Multilamellar vesicle
MPS: - Mononuclear phagocytic system
NP: - Nanoparticle
OLV: - Oligolamellar vesicle
PA: - Phosphatidic acid
PC: - Phosphatidylcholine
PE: - Phosphatidylethanolamine
PEG: - Polyethylene glycol
PEG-DSPE: - Polyethylene glycol-distearoylphosphatidylethanolamine
pDNA: - Plasmid DNA
siRNA: - Small interfering RNA
Tc: - Phase transition temperature
TEM: - Transmission electron microscopy
